# 1-Methyl-3-trifluoro­methyl-1*H*-pyrazol-5-ol

**DOI:** 10.1107/S1600536809035624

**Published:** 2009-09-09

**Authors:** Jia-ying Xu, Wei-hua Cheng, Jin-long Yan, Gui-xiang Quan

**Affiliations:** a Department of Applied Chemistry, College of Chemical and Biological Engineering, Yancheng Institute of Technology, Yinbing Road No. 9 Yancheng, Yancheng 224051, People’s Republic of China; b Department of Chemical Engineering, Yancheng College of Textile Technology, Liberation Road S. No. 265 Yancheng, Yancheng 224005, People’s Republic of China

## Abstract

In the title compound, C_5_H_5_F_3_N_2_O, the F atoms are disordered over two sets of sites in a 0.64 (3):0.36 (3) ratio. In the crystal structure, O—H⋯N hydrogen bonds link the mol­ecules into chains and a short C—H⋯F contact also occurs.

## Related literature

For background to fluorinated heterocycles, see: Marcos & Martins (2003 ▶). For bond-length data, see: Allen *et al.* (1987 ▶).
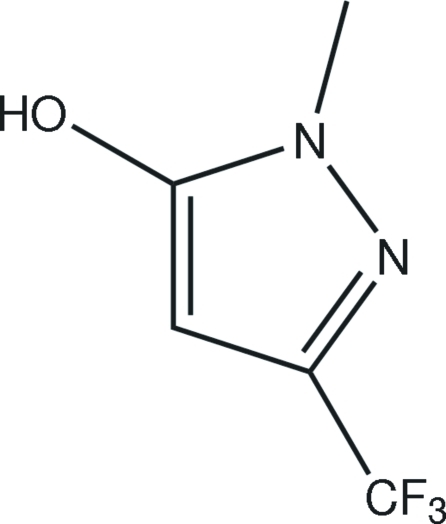


## Experimental

### 

#### Crystal data

C_5_H_5_F_3_N_2_O
*M* *_r_* = 166.11
Monoclinic, 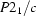

*a* = 7.5500 (15) Å
*b* = 8.3530 (17) Å
*c* = 11.371 (2) Å
β = 104.72 (3)°
*V* = 693.6 (2) Å^3^
*Z* = 4
Mo *K*α radiation
μ = 0.17 mm^−1^
*T* = 293 K
0.20 × 0.10 × 0.10 mm

#### Data collection

Enraf–Nonius CAD-4 diffractometer
Absorption correction: ψ scan (North *et al.*, 1968 ▶) *T*_min_ = 0.968, *T*_max_ = 0.984
1357 measured reflections
1259 independent reflections
856 reflections with *I* > 2σ(*I*)
*R*_int_ = 0.041
3 standard reflections every 200 reflections intensity decay: 1%

#### Refinement

*R*[*F* ^2^ > 2σ(*F* ^2^)] = 0.050
*wR*(*F* ^2^) = 0.168
*S* = 1.00
1259 reflections
130 parameters
36 restraints
H-atom parameters constrained
Δρ_max_ = 0.20 e Å^−3^
Δρ_min_ = −0.21 e Å^−3^

### 

Data collection: *CAD-4 EXPRESS* (Enraf–Nonius, 1994 ▶); cell refinement: *CAD-4 EXPRESS*; data reduction: *XCAD4* (Harms & Wocadlo, 1995 ▶); program(s) used to solve structure: *SHELXS97* (Sheldrick, 2008 ▶); program(s) used to refine structure: *SHELXL97* (Sheldrick, 2008 ▶); molecular graphics: *SHELXTL* (Sheldrick, 2008 ▶) and *ORTEP-3* (Farrugia, 1997 ▶); software used to prepare material for publication: *PLATON* (Spek, 2009 ▶).

## Supplementary Material

Crystal structure: contains datablocks global, I. DOI: 10.1107/S1600536809035624/hb5087sup1.cif

Structure factors: contains datablocks I. DOI: 10.1107/S1600536809035624/hb5087Isup2.hkl

Additional supplementary materials:  crystallographic information; 3D view; checkCIF report

## Figures and Tables

**Table 1:** Hydrogen-bond geometry (Å, °)

*D*—H⋯*A*	*D*—H	H⋯*A*	*D*⋯*A*	*D*—H⋯*A*
O—H0*A*⋯N1^i^	0.85	1.85	2.698 (3)	176
C5—H5*B*⋯F3′^ii^	0.96	2.55	3.185 (12)	124

Symmetry codes: (i) 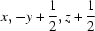; (ii) 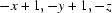.

**Fig. 1.**
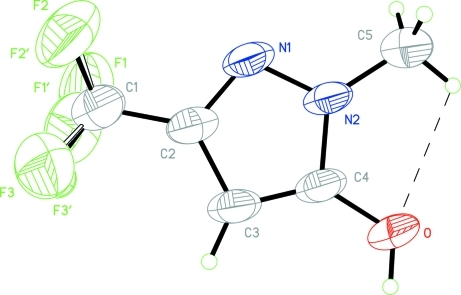
The molecular structure of (I).

**Fig. 2.**
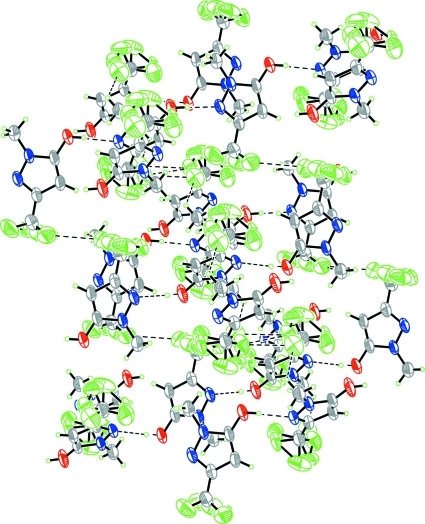
A partial packing diagram of (I). Hydrogen bonds are shown as dashed lines.

## supplementary crystallographic information

### Comment

As part of the ongoing study of polyfluorinated heterocycles (Marcos & Martins, 2003), we report herein the crystal structure of the title compound.

In the molecule of the title compound (Fig 1), the bond lengths (Allen *et al.*,  1987) and angles are within normal ranges. Ring A (C4-C9) is, of course, planar. The intramolecular C-H···O hydrogen bond (Table 1) results in the formation of a five-membered ring B (O1/C1-C3/H1A), having envelope conformation with C2 atom displaced by -0.668 (3) Å from the plane of the other ring atoms.

In the crystal structure, intermolecular O—H···N hydrogen bonds (Table 1) link the molecules (Fig. 2), in which they may be effective in the stabilization of the structure.

### Experimental

4,4-Diethoxy-1,1,1-trifluorobut-3-en-2-one (2 mmol) was added to a stirred solution of hydrazine (2.2 mmol) at room temperature in ethanol (15 ml). The mixture was stirred under reflux for 24 h. The solvent was evaporated and to the residue was added H2O (10 ml) and the organic phase were extract with dichloromethane (15 ml). The organic extract was dried (Na_2_SO_4_) and the solvent was removed under reduced pressure to obtain the title compound (yield; 25%, m.p. 446 K). Colourless blocks of (I) were obtained by slow evaporation of an ethyl acetate solution.

### Refinement

H atoms were positioned geometrically, with C—H = 0.93, 0.97 and 0.96 Å for aromatic, methylene and methyl H, respectively, and constrained to ride on their parent atoms, with U_iso_(H) = xU_eq_(C), where x = 1.5 for methyl H and x = 1.2 for all other H atoms.

### Crystal data

**Table:**

C_5_H_5_F_3_N_2_O	*F*(000) = 336
*M**_r_* = 166.11	*D*_x_ = 1.591 Mg m^−^^3^
Monoclinic, *P*2_1_/*c*	Melting point: 446 K
Hall symbol: -P 2ybc	Mo *K*α radiation, λ = 0.71073 Å
*a* = 7.5500 (15) Å	Cell parameters from 25 reflections
*b* = 8.3530 (17) Å	θ = 9–13°
*c* = 11.371 (2) Å	µ = 0.17 mm^−^^1^
β = 104.72 (3)°	*T* = 293 K
*V* = 693.6 (2) Å^3^	Block, colourless
*Z* = 4	0.20 × 0.10 × 0.10 mm

### Data collection

**Table:**

Enraf–Nonius CAD-4 diffractometer	856 reflections with *I* > 2σ(*I*)
Radiation source: fine-focus sealed tube	*R*_int_ = 0.041
graphite	θ_max_ = 25.3°, θ_min_ = 2.8°
ω/2θ scans	*h* = 0→9
Absorption correction: ψ scan (North *et al.*, 1968)	*k* = 0→10
*T*_min_ = 0.968, *T*_max_ = 0.984	*l* = −13→13
1357 measured reflections	3 standard reflections every 200 reflections
1259 independent reflections	intensity decay: 1%

### Refinement

**Table:**

Refinement on *F*^2^	Secondary atom site location: difference Fourier map
Least-squares matrix: full	Hydrogen site location: inferred from neighbouring sites
*R*[*F*^2^ > 2σ(*F*^2^)] = 0.050	H-atom parameters constrained
*wR*(*F*^2^) = 0.168	*w* = 1/[σ^2^(*F*_o_^2^) + (0.1*P*)^2^ + 0.12*P*] where *P* = (*F*_o_^2^ + 2*F*_c_^2^)/3
*S* = 1.00	(Δ/σ)_max_ < 0.001
1259 reflections	Δρ_max_ = 0.20 e Å^−^^3^
130 parameters	Δρ_min_ = −0.21 e Å^−^^3^
36 restraints	Extinction correction: *SHELXL97* (Sheldrick, 2008), Fc^*^=kFc[1+0.001xFc^2^λ^3^/sin(2θ)]^-1/4^
Primary atom site location: structure-invariant direct methods	Extinction coefficient: 0.062 (13)

### Special details

**Table:**

Geometry. All esds (except the esd in the dihedral angle between two l.s. planes) are estimated using the full covariance matrix. The cell esds are taken into account individually in the estimation of esds in distances, angles and torsion angles; correlations between esds in cell parameters are only used when they are defined by crystal symmetry. An approximate (isotropic) treatment of cell esds is used for estimating esds involving l.s. planes.
Refinement. Refinement of F^2^ against ALL reflections. The weighted R-factor wR and goodness of fit S are based on F^2^, conventional R-factors R are based on F, with F set to zero for negative F^2^. The threshold expression of F^2^ > 2sigma(F^2^) is used only for calculating R-factors(gt) etc. and is not relevant to the choice of reflections for refinement. R-factors based on F^2^ are statistically about twice as large as those based on F, and R- factors based on ALL data will be even larger.

### Fractional atomic coordinates and isotropic or equivalent isotropic displacement parameters (Å^2^)

**Table:**

	*x*	*y*	*z*	*U*_iso_*/*U*_eq_	Occ. (<1)
O	0.6146 (3)	0.1822 (3)	0.22551 (16)	0.0812 (8)	
H0A	0.5735	0.1988	0.2874	0.097*	
N1	0.4722 (3)	0.2582 (3)	−0.08398 (18)	0.0562 (7)	
C1	0.1757 (5)	0.3877 (5)	−0.1412 (3)	0.0701 (9)	
N2	0.5857 (3)	0.2020 (3)	0.02028 (18)	0.0549 (7)	
C2	0.3341 (4)	0.3246 (3)	−0.0489 (2)	0.0540 (8)	
C3	0.3563 (4)	0.3122 (4)	0.0765 (2)	0.0609 (8)	
H3A	0.2783	0.3492	0.1220	0.073*	
C4	0.5184 (4)	0.2333 (4)	0.1169 (2)	0.0585 (8)	
C5	0.7551 (4)	0.1216 (5)	0.0201 (3)	0.0697 (9)	
H5A	0.8043	0.0723	0.0978	0.105*	
H5B	0.8414	0.1981	0.0046	0.105*	
H5C	0.7323	0.0411	−0.0422	0.105*	
F1	0.0666 (15)	0.2656 (12)	−0.1980 (10)	0.117 (3)	0.64 (3)
F2	0.2287 (15)	0.4689 (17)	−0.2294 (11)	0.107 (3)	0.64 (3)
F3	0.057 (2)	0.458 (3)	−0.0997 (12)	0.093 (5)	0.36 (3)
F3'	0.1113 (17)	0.5237 (14)	−0.0992 (8)	0.103 (2)	0.64 (3)
F2'	0.202 (2)	0.412 (2)	−0.2461 (11)	0.077 (4)	0.36 (3)
F1'	0.0325 (19)	0.309 (3)	−0.157 (2)	0.105 (5)	0.36 (3)

### Atomic displacement parameters (Å^2^)

**Table:**

	*U*^11^	*U*^22^	*U*^33^	*U*^12^	*U*^13^	*U*^23^
O	0.0761 (14)	0.143 (2)	0.0277 (11)	0.0173 (14)	0.0192 (9)	0.0057 (11)
N1	0.0633 (14)	0.0805 (17)	0.0266 (11)	−0.0061 (12)	0.0150 (10)	−0.0014 (10)
C1	0.075 (2)	0.089 (2)	0.0477 (18)	0.003 (2)	0.0183 (16)	0.0058 (16)
N2	0.0585 (13)	0.0824 (16)	0.0266 (11)	−0.0018 (12)	0.0162 (9)	−0.0031 (10)
C2	0.0632 (17)	0.0676 (18)	0.0346 (13)	−0.0069 (14)	0.0190 (12)	−0.0019 (12)
C3	0.0642 (17)	0.090 (2)	0.0338 (14)	−0.0021 (16)	0.0221 (12)	−0.0070 (13)
C4	0.0606 (17)	0.090 (2)	0.0277 (13)	−0.0054 (16)	0.0164 (12)	−0.0050 (13)
C5	0.0675 (18)	0.100 (2)	0.0454 (16)	0.0037 (18)	0.0219 (14)	−0.0017 (15)
F1	0.096 (4)	0.120 (4)	0.104 (5)	−0.001 (3)	−0.033 (3)	−0.017 (3)
F2	0.118 (4)	0.122 (6)	0.076 (4)	−0.003 (4)	0.016 (3)	0.042 (4)
F3	0.088 (6)	0.120 (10)	0.074 (4)	0.036 (6)	0.027 (4)	−0.009 (6)
F3'	0.112 (5)	0.099 (5)	0.095 (3)	0.028 (4)	0.020 (3)	0.000 (3)
F2'	0.091 (6)	0.114 (8)	0.030 (3)	0.019 (5)	0.024 (3)	0.011 (4)
F1'	0.073 (5)	0.114 (9)	0.110 (8)	−0.029 (5)	−0.008 (5)	0.014 (6)

### Geometric parameters (Å, °)

**Table:**

O—C4	1.334 (3)	C1—C2	1.474 (4)
O—H0A	0.8501	N2—C4	1.348 (3)
N1—C2	1.328 (3)	N2—C5	1.445 (4)
N1—N2	1.358 (3)	C2—C3	1.397 (4)
C1—F1'	1.238 (13)	C3—C4	1.363 (4)
C1—F3	1.257 (12)	C3—H3A	0.9300
C1—F2'	1.274 (12)	C5—H5A	0.9600
C1—F2	1.352 (10)	C5—H5B	0.9600
C1—F1	1.366 (9)	C5—H5C	0.9600
C1—F3'	1.369 (9)		
			
C4—O—H0A	119.2	F1—C1—C2	110.6 (5)
C2—N1—N2	104.6 (2)	F3'—C1—C2	110.2 (5)
F1'—C1—F3	68 (2)	C4—N2—N1	111.1 (2)
F1'—C1—F2'	106.6 (10)	C4—N2—C5	127.5 (2)
F3—C1—F2'	124.7 (11)	N1—N2—C5	121.5 (2)
F1'—C1—F2	124.7 (8)	N1—C2—C3	112.1 (3)
F3—C1—F2	114.7 (14)	N1—C2—C1	119.5 (2)
F2'—C1—F2	23.0 (8)	C3—C2—C1	128.2 (3)
F1'—C1—F1	30.3 (9)	C4—C3—C2	104.2 (2)
F3—C1—F1	97.0 (17)	C4—C3—H3A	127.9
F2'—C1—F1	84.1 (8)	C2—C3—H3A	127.9
F2—C1—F1	106.3 (6)	O—C4—N2	117.6 (3)
F1'—C1—F3'	96.6 (18)	O—C4—C3	134.3 (2)
F3—C1—F3'	29.9 (8)	N2—C4—C3	108.1 (2)
F2'—C1—F3'	110.2 (11)	N2—C5—H5A	109.5
F2—C1—F3'	92.4 (11)	N2—C5—H5B	109.5
F1—C1—F3'	124.0 (11)	H5A—C5—H5B	109.5
F1'—C1—C2	115.8 (7)	N2—C5—H5C	109.5
F3—C1—C2	115.2 (6)	H5A—C5—H5C	109.5
F2'—C1—C2	115.7 (7)	H5B—C5—H5C	109.5
F2—C1—C2	111.6 (5)		
			
C2—N1—N2—C4	0.0 (3)	F2'—C1—C2—C3	168.0 (11)
C2—N1—N2—C5	−179.4 (3)	F2—C1—C2—C3	143.2 (8)
N2—N1—C2—C3	−0.1 (3)	F1—C1—C2—C3	−98.6 (8)
N2—N1—C2—C1	−175.2 (3)	F3'—C1—C2—C3	42.1 (8)
F1'—C1—C2—N1	108.2 (15)	N1—C2—C3—C4	0.1 (3)
F3—C1—C2—N1	−175.6 (14)	C1—C2—C3—C4	174.7 (3)
F2'—C1—C2—N1	−17.7 (11)	N1—N2—C4—O	178.9 (3)
F2—C1—C2—N1	−42.5 (8)	C5—N2—C4—O	−1.7 (5)
F1—C1—C2—N1	75.7 (8)	N1—N2—C4—C3	0.0 (3)
F3'—C1—C2—N1	−143.6 (7)	C5—N2—C4—C3	179.4 (3)
F1'—C1—C2—C3	−66.1 (16)	C2—C3—C4—O	−178.7 (4)
F3—C1—C2—C3	10.1 (15)	C2—C3—C4—N2	0.0 (3)

### Hydrogen-bond geometry (Å, °)

**Table:**

*D*—H···*A*	*D*—H	H···*A*	*D*···*A*	*D*—H···*A*
O—H0A···N1^i^	0.85	1.85	2.698 (3)	176
C5—H5B···F3'^ii^	0.96	2.55	3.185 (12)	124

Symmetry codes: (i) *x*, −*y*+1/2, *z*+1/2; (ii) −*x*+1, −*y*+1, −*z*.
